# Satisfaction des patients opérés en chirurgie ORL au Centre hospitalier universitaire Sylvanus Olympio de Lomé (Togo)

**DOI:** 10.48327/mtsi.v5i1.2025.638

**Published:** 2025-01-29

**Authors:** Essobiziou AMANA, Winga FOMA, Guemessou NASSOU, Gérémie ANANIDJIN, Bathokédéou AMANA

**Affiliations:** Service d'oto-rhino-laryngologie, Centre hospitalier universitaire, Sylvanus Olympio de Lomé, Togo

**Keywords:** Satisfaction, Consultation, ORL, Bloc opératoire, Thyroïdectomie, Lomé, Togo, Afrique subsaharienne, Satisfaction, Consultation, ENT, Operating room, Thyroidectomy, Lomé, Togo, Sub-Saharan Africa

## Abstract

**Objectif:**

la satisfaction des patients opérés dans un service d'oto-rhino-laryngologie. Étude réalisée au CHU Sylvanus Olympio de Lomé, de la consultation à la prise en charge chirurgicale.

**Patients et méthodes:**

Étude transversale allant du 1er décembre 2022 au 30 novembre 2023, soit sur une période d'un an. Étaient concernés par l'étude les patients ou les accompagnants de patients de moins de 18 ans, ayant donné leur consentement pour l'enquête, ayant été vus par l'équipe chirurgicale et opérés dans le service au cours de la période d'étude. Les paramètres évalués par auto-questionnaires fermés étaient en rapport avec la satisfaction depuis l'accueil jusqu'au diagnostic, du bilan pré-opératoire jusqu'à la veille de l'intervention, du jour de l'intervention jusqu'à la fin de l'hospitalisation et enfin en rapport avec les formalités de sortie de l'hôpital. L'analyse et le traitement des données ont été faits au moyen du logiciel Epi info 7.2.5.0.

**Résultats:**

Cent douze personnes dont quinze accompagnants de patients, ont répondu aux critères de l'enquête, soit 70,4 %. L'âge moyen des participants à l'enquête était de 41 ans avec des extrêmes de 19 et 76 ans. Les participants avaient un niveau secondaire d'instruction dans 59,5 % des cas. C'étaient des commerçants et des ménagères dans respectivement 26,8 % et 24,1 % des cas. La thyroïdectomie était le type d'intervention le plus pratiqué, soit 43,7 % des cas. De l'accueil jusqu'au diagnostic, l'accueil dans le service était ressenti comme satisfaisant dans 66,1 % des cas et très mauvais dans 12,5 % des cas. Du bilan pré-opératoire jusqu'à la veille de l'intervention, les formalités de ce bilan et l'achat des ordonnances étaient jugés mauvais dans respectivement 33,9 % cas et 40,2 % des cas. Du jour de l'intervention jusqu'à la sortie, la qualité des sanitaires et de la salle d'hospitalisation était mauvaise dans respectivement 44,6 % et 54,4 % des cas. Les participants étaient satisfaits de la qualité des visites (58,9 %), du comportement du personnel au bloc (30,4 %), de l'information sur le suivi et de l'acte chirurgical dans tous les cas.

**Conclusion:**

La non-satisfaction des patients et de leurs accompagnants existe à tous les niveaux, administratifs et médicaux. Des efforts supplémentaires restent à fournir dans notre service pour améliorer la qualité des soins.

## Abstract (en)

**Objective:**

To evaluate the satisfaction of patients surgically treated in a Otolaryngology Department in a developing country. This survey was caried out from consultation to surgical management in the Sylvanus Olympio University Hospital in Lomé.

**Patients and Methods:**

Cross-sectional study from December 1, 2022 to November 30, 2023, a period of one year. People included were the patients or relatives for under 18 years of age who gave their consent, seen by the surgical team and operated on in the department during this period. The parameters assessed by a closed self-administered questionnaire were related to satisfaction from admission to diagnosis, from preoperative evaluation to the day before surgery, from the day of surgery to the end of hospitalization, and finally to discharge formalities. The data were analyzed and processed using Epi info 7.2.5.0 software.

**Results:**

One hundred and twelve individuals, including fifteen relatives, met the survey criteria (70.4%). The mean age was 41 years, with extremes of 19 and 76 years. The participants had a secondary education level in 59.5% of the cases. They were shopkeepers and housewives in 26.8% and 24.1% of cases, respectively. Thyroidectomy was the most common type of surgery, performed in 43.7% of cases. From admission to diagnosis, 66.1% of patients found the service satisfactory, while 12.5% found it very poor. From the preoperative examination to the day before surgery, this examination and the purchase of prescriptions were judged to be poor in 33.9% and 40.2% of cases, respectively. From the day of the operation to discharge, the quality of the sanitary facilities and of the hospital ward was poor in 44.6% and 54.4% of cases, respectively. Participants were satisfied with the quality of visits (58.9%), behavior of medical-surgical team in the operating room (30.4%), follow-up information and surgical procedure in all cases.

**Conclusion:**

Dissatisfaction of patients and their relatives exists at all levels, administrative and medical. More efforts need to be done in our department to improve the quality of care.

## Introduction

Recueillir la satisfaction des patients est de nos jours un des moyens d’évaluer la perception de la qualité des soins. Cette évaluation, bien que courante dans les pays développés depuis bien longtemps [1, 2], reste encore à l’état embryonnaire dans les pays en voie de développement tels que les pays africains. Elle peut se faire par le recueil des plaintes, l'utilisation des questionnaires à la sortie ou par une enquête spécifique [3].

Dans le but d'améliorer la pratique chirurgicale dans notre service hospitalier, et plus généralement dans notre pays, les auteurs ont initié cette étude qui n'est que préliminaire, dont l'objectif était d’évaluer la satisfaction des patients opérés, plus précisément depuis leur consultation jusqu’à leur sortie de l'hôpital.

## Patients et méthodes

Le service d’ORL et de chirurgie cervico-maxillo-faciale du CHU Sylvanus Olympio a servi de cadre d’étude. Il s'est agi d'une étude transversale d'un an, allant du 1^er^ décembre 2022 au 30 novembre 2023. Ont été inclus dans l’étude les patients de plus de 18 ans ou les accompagnants des patients de moins de 18 ans, ayant donné leur consentement pour l'enquête, ayant consulté dans le service, vus par le *staff* chirurgical et opérés dans le service au cours de la période d’étude. Le *staff* chirurgical consistait en une réunion au cours de laquelle les dossiers des patients à opérer étaient passés en revue en présence du patient et de son accompagnant d'une part, et des enseignants universitaires accompagnés des médecins spécialistes et des médecins en cours de spécialisation, d'autre part. Les patients sélectionnés sont ceux qui ont directement consulté dans le service pour une pathologie ORL et n'ayant pas d'antécédents d'hospitalisation.

N'ont pas été inclus les patients n'ayant pas donné leur consentement, les patients opérés en urgence et ceux ayant un antécédent d'hospitalisation. Les paramètres évalués ont été l’âge, le sexe, la profession, le niveau d’étude ainsi que les questionnaires en rapport avec la satisfaction depuis l'accueil jusqu'au diagnostic, du bilan pré-opératoire jusqu’à la veille de l'intervention, du jour de l'intervention jusqu’à la fin de l'hospitalisation et enfin en rapport avec les formalités de sortie de l'hôpital.

L'appréciation de la satisfaction a été évaluée en utilisant le questionnaire Saphora (Très satisfaisant, satisfaisant, mauvais, très mauvais) [8].

La fiche d'enquête a été préalablement expliquée aux patients.

Cette fiche a été ensuite remplie par le patient ou l'accompagnant à la sortie ou au contrôle postopératoire. L'analyse et le traitement des données ont été faits au moyen du logiciel Epi info 7.2.5.0

## Résultats

Sur 159 interventions chirurgicales programmées durant l'année de l’étude, un effectif de 112 a répondu aux critères et à l'enquête, soit un taux de participation de 70,4 %. Quarante-sept patients ou accompagnants de patients (soit 29,6 %) n'ont pas, de façon délibérée et selon leur convenance personnelle, répondu à l'enquête.

**Figure 1:**
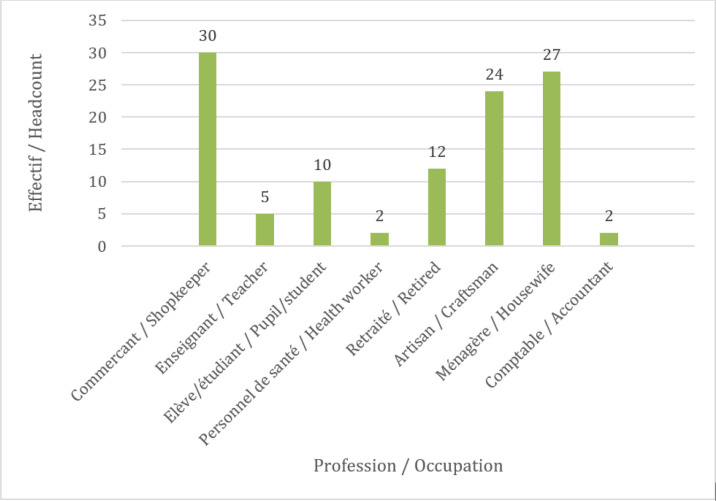
Effectifs des professions des participants

**Figure 2:**
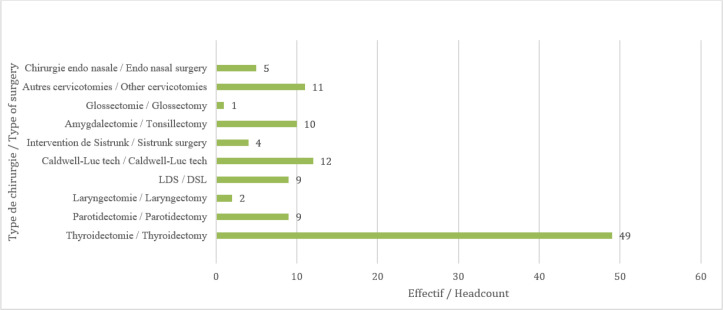
Répartition des participants selon le type de chirurgie

L’âge moyen était de 40,2± 14,7 ans avec les extrêmes de 19 ans et 76 ans. Le sexe féminin concernait 85 cas soit un sexe ratio de 0,3. Les commerçants constituaient 30 cas, suivis des ménagères dans 27 cas (Fig. 1). Les artisans et les ménagères ont trouvé satisfaisantes toutes les procédures dans 87 % des cas, les commerçants dans 82 % des cas, le personnel de santé, les comptables et les retraités dans 46 % des cas. Pour ce qui est du niveau d'instruction des participants, 68 cas étaient du niveau secondaire, 30 du niveau supérieur et 14 du niveau primaire. Le type d'intervention était une thyroïdectomie dans 49 cas (Fig. 2).

De l'accueil jusqu'au diagnostic, les patients étaient satisfaits de l'accueil dans 66,1 % des cas et ont trouvé mauvaise l'attente lors de la consultation dans 9,8 % des cas (Tableau I).

**Tableau I:** Satisfaction de l'accueil jusqu'au diagnostic

	Très satisfait	Satisfait	Mauvais	Très mauvais
Accueil dans le service	0	74 (66,1 %)	24 (21,4 %)	14 (12,5 %)
Attente lors de la consultation	24 (21,5 %)	77 (68,7 %)	11 (9,8 %)	0
Information sur la pathologie	34 (30,3 %)	74 (66,1 %)	4 (3,6 %)	0

Du bilan préopératoire jusqu’à la veille de l'intervention, les patients étaient satisfaits de leur passage devant le *staff* chirurgical et de la qualité des informations médicales dans respectivement 66,1 % et 71,4 % des cas (Tableau II).

**Tableau II:** Satisfaction du bilan pré-opératoire (BPO) jusqu'à la veille de l'intervention

	Très satisfait	Satisfait	Mauvais	Très mauvais
Formalités pour le BPO	0	74 (66,1 %)	38 (33,9 %)	0
Consultation pré anesthésique	10 (8,9 %)	88 (78,5 %)	14(12,6 %)	0
Passage au staff chirurgical	21 (18,7 %)	74 (66,1 %)	17(15,2 %)	0
Qualité des informations sur l'intervention	26 (23,2 %)	80 (71,5 %)	6 (5,3 %)	0
Achat des ordonnances pré-opératoires	14 (12,5 %)	53 (47,3 %)	45 (40,2 %)	0
Accueil la veille de l'intervention	18 (16,1 %)	85 (75,9 %)	9 (8 %)	0
Qualité de la salle d'hospitalisation	2(1,8 %)	24 (21,4 %)	61 (54,5 %)	25 (22,3 %)
Qualité des sanitaires	5 (4,5 %)	32 (28,6 %)	50 (44,6 %)	25 (22,3 %)

Du jour de l'intervention jusqu’à la fin de l'hospitalisation, 16,1 % des cas étaient très satisfaits du comportement de l'équipe médico-chirurgicale au bloc et 17,9 % des cas l'ont trouvé mauvais (Tableau III).

**Tableau III:** Satisfaction du jour de l'intervention jusqu'à la fin de l'hospitalisation

	Très satisfait	Satisfait	Mauvais	Très mauvais
Identification du personnel	8(7,2 %)	38 (33,9 %)	66 (58,9 %)	0
Respect de l'intimité	13 (11,6 %)	72 (64,3 %)	27 (24,1 %)	0
Respect de la confidentialité des informations	18 (16,1 %)	80 (71,4 %)	12 (10,7 %)	2 (1,8 %)
Délai d'attente avant de rentrer au bloc	16 (14,3 %)	90 (80,4 %)	6 (5,3 %)	0
Comportement du personnel au bloc	18 (16 %)	54 (48,2 %)	20 (17,9 %)	20 (17,9 %)
Qualité de la salle d'hospitalisation	8 (7,1 %)	29 (25,9 %)	61 (54,5 %)	14 (12,5 %)
Qualité des visites	32 (28,6 %)	66 (58,9 %)	14 (12,5 %)	0
Attitude des infirmiers	24 (21,4 %)	66 (58,9 %)	22 (19,7 %)	0
Attitude des techniciens de surface	0	77 (68,7 %)	29 (25,9 %)	6(5,4 %)
Attitude de l'équipe de garde	19 (17 %)	80 (71,4 %)	13 (11,6 %)	0

Pour les procédures de sortie de l'hôpital, les patients étaient satisfaits des informations données sur l'ordonnance de sortie et sur le suivi post-opératoire dans respectivement 71,4 % et 80,4 % des cas (Tableau IV).

**Tableau IV:** Satisfaction du jour de l'intervention jusqu'à la fin de l'hospitalisation

	Très satisfait	Satisfait	Mauvais	Très mauvais
Information sur l'ordonnance de sortie	27 (24,1 %)	80 (71,2 %)	5 (4,47 %)	0
Information sur le suivi	21 (18,7 %)	90 (80,4 %)	1 (0,9 %)	0
Formalités administratives	18 (16,1 %)	90 (80,3 %)	4 (3,6 %)	0
Appréciation sur la chirurgie	40 (35,7 %)	72 (64,3 %)	0	0

## Discussion

Un des biais de notre étude est l'absence et la méconnaissance de la charte du malade hospitalisé, qui entraînent des fausses appréciations du service rendu par le personnel médical par le patient qui n'a aucune référence pour juger. On assiste à des fausses satisfactions et à des fausses insatisfactions. D'autres biais sont la santé retrouvée qui a pu entraîner des appréciations positives excessives. Les patients venus des zones rurales qui ne posent généralement aucune exigence sont parfois satisfaits de tout. Ces biais sont communs à la plupart des études dans la littérature [10]. Les autres limites de l’étude sont le faible échantillonnage, l'utilisation du français dans la fiche d'enquête nécessitant d'amples explications aux enquêtés ayant un faible niveau en français, le désir d'abstention de certains, pensant apporter un faux jugement pouvant nuire à la structure sanitaire.

Plus du tiers des patients n’était pas satisfait de l'accueil dans le service, en raison, principalement, du peu d'amabilité du personnel soignant. L'accueil est plus qu'un acte banal de la vie quotidienne et l'arrivée d'un malade dans un service de soins est particulière. C'est pour lui un temps fort, un moment où il est sensible et vulnérable et où il a besoin de se raccrocher à quelqu'un [10]. Les séances de formation et d'information organisées par le syndicat des praticiens hospitaliers du Togo permettront de remédier à ce défaut. Il est important de bien accueillir les patients car le traitement commence dès l'accueil.

La procédure pour honorer les ordonnances préopératoires a été ressentie comme mauvaise dans 40,2 % des cas. En effet, c'est le patient lui-même qui va d'officine en officine pour obtenir les remèdes et matériels prescrits, les premières ne possédant souvent pas toute la gamme prescrite. Des kits préformés de médicaments d'anesthésie et de chirurgie pour les interventions standard pourraient remédier à cette procédure difficile. La Fondation nationale pour la sécurité des patients aux États-Unis a défini la sécurité des patients comme l’évitement, la prévention et l'amélioration des résultats indésirables ou des blessures découlant des processus de soins de santé [4]. Le passage au *staff* chirurgical s'inscrit dans cette ligne. Il a été apprécié, jugé très satisfaisant par 80 % des patients. Pour minimiser les incidents et accidents peropératoires, certains auteurs recommandent un *staff* chirurgical afin de discuter du dossier avant l'intervention chirurgicale [9]. Dans notre cas, la programmation de tous les patients au *staff* chirurgical est systématique sauf en cas d'urgence. Il permet à l’équipe de confirmer le diagnostic, d'expliquer une fois encore au patient le geste chirurgical, de former les médecins en spécialisation. Certains patients n'ont pas apprécié leur passage devant ce *staff* chirurgical, du fait de l'effectif impressionnant de médecins et de la durée d'attente avant la rencontre.

Les patients ont été satisfaits du respect de la confidentialité et de la qualité des informations sur la pathologie dans 71,4 % des cas, ce qui est en conformité avec le Code de santé publique togolais qui stipule respectivement, en ses articles 192 et 196, que « *le praticien est tenu au secret professionnel* (…) », et que « *tout patient est libre de s'adresser au praticien de son choix. Celui-ci doit l’éclairer sur son état de santé, les soins qu'il doit recevoir et les conséquences que ceux-ci peuvent entraîner pour lui* » [6]. Malgré les conditions d'hospitalisation dans le service d’ORL, à savoir une hospitalisation en salle commune avec des lits non séparés par une barrière, et également l'effectif des patients par consultation, il est possible et nécessaire de respecter le secret professionnel et d'expliquer en détails le diagnostic et la prise en charge du patient. Cette procédure est particulièrement importante dans le cadre d'une prise en charge chirurgicale.

Le comportement du personnel au bloc a été jugé mauvais et très mauvais dans 17,9 % des cas chacun. En effet, le bloc opératoire contenait, en dehors du personnel du bloc du service, des étudiants stagiaires en licence en instrumentation chirurgicale, en anesthésie et réanimation et des médecins en diplôme d’études spécialisées. Un effectif important peut créer une peur chez le patient. De plus, en pré induction la communication ouverte au bloc opératoire consistant à signaler le respect de certaines règles, à protester contre un comportement inapproprié et à faire des remarques, effraie le patient installé sur la table opératoire [5, 7]. Les bavardages et les remarques inappropriées entre personnels, sont mal perçues par le patient installé sur la table opératoire lors de l'induction de l'anesthésie.

## Conclusion

Il ressort de cette étude que la non-satisfaction est à tous les niveaux, administratif et médical. Nous recommandons à la fin de cette étude le respect scrupuleux du code de déontologie et d’éthique médicale ainsi que de la charte du patient hospitalisé, afin de satisfaire tout patient admis dans une structure sanitaire. Une étude prospective sera menée sur un échantillon plus grand et avec la prise en compte de la charte du patient. Cela permettra une évaluation plus approfondie de la satisfaction de la qualité des soins dans notre service.

## Source de financement

Les auteurs ne déclarent aucune source de financement.

## Contribution des auteurs

Essobiziou AMANA, Winga FOMA, et Guemessou NASSOU : collecte des données et rédaction de l'article. Gérémie ANANIDJIN, Bathokédéou AMANA : relecture de l'article.

## Conflit d'intérêt

Les auteurs déclarent ne pas avoir de liens d'intérêts.
