# Supplementary material for: Patients’ satisfaction in ENT surgery at the Sylvanus Olympio University Hospital in Lomé (Togo)
Source: Med Trop Sante Int. 2025 Jan 29;5(1):mtsi.v5i1.2025.638. [Article in French] doi: 10.48327/mtsi.v5i1.2025.638 (PMC12001988; doi:10.48327/mtsi.v5i1.2025.638)
Supplement: Related texts

# Research article: Patients’ satisfaction in ENT surgery at the Sylvanus Olympio University Hospital in Lomé (Togo)

## Introduction

Measuring patient satisfaction is one of the modern ways of assessing perceived quality of care. Although this type of assessment has long been commonplace in developed countries [1, 2], it is still in its infancy in developing countries such as Africa. It can be done by collecting complaints, using discharge questionnaires, or through a specific survey [3].

With the aim of improving surgical practice in our department and more generally in our country, the authors initiated this study, which is only preliminary, with the aim of evaluating the satisfaction of operated patients, more precisely from their consultation to their discharge from the hospital.

## Patients and methods

The Department of Otolaryngology and Cervicomaxillofacial Surgery of CHU Sylvanus Olympio served as the study setting. This was a one-year cross-sectional study, from December 1, 2022 to November 30, 2023. Patients over the age of 18 years or relatives for the < 18 years old were included if they consented to the survey, were consulted in the department, were seen by the surgical staff, and underwent surgery in the department during the study period. The surgical staff consisted of a meeting during which the files of the patients to be operated on were reviewed in the presence of the patient and his companion, on the one hand, and the university lecturers accompanied by specialists and doctors undergoing specialization, on the other. The patients selected were those who had consulted the department directly for ENT pathology and who did not have a history of previous hospitalization.

Patients who did not give consent, those who underwent emergency surgery, and those with a history of hospitalization were not included.

The parameters evaluated were age, sex, occupation, education level, and questionnaires on satisfaction from admission to diagnosis, from preoperative examination to the day before surgery, from the day of surgery to the end of hospitalization, and finally in relation to hospital discharge formalities.

Satisfaction was assessed using the Saphora questionnaire (very satisfactory, satisfactory, poor, very poor) [8].

The questionnaire was explained to the patients beforehand.

This form was then filled out by the patient or the accompanying person at discharge or at the postoperative check-up. Data were analyzed and processed using Epi info 7.2.5.0 software.

## Results

Of 159 surgical procedures scheduled during the study year, a total of 112 patients met the criteria and completed the survey, resulting in a participation rate of 70.4%. Forty-seven patients or their relatives (29.6%) deliberately chose not to complete the survey.

The mean age was 40.2±14.7 years, with extremes of 19 and 76 years. Females accounted for 85 cases, with a sex ratio of 0.3. Shopkeepers accounted for 30 cases, followed by housewives with 27 cases (Fig. 1). Artisans and housewives found all procedures satisfactory in 87% of cases, shopkeepers in 82% of cases, and health workers, accountants and pensioners in 46% of cases.

**Figure 1:** Number of participants by occupation

Regarding the educational level of the participants, 68 cases were at the secondary level, 30 at the tertiary level, and 14 at the primary level. Thyroidectomy was performed in 49 cases (Fig. 2).

**Figure 2:** Distribution of participants by type of surgery

From reception to diagnosis, patients were satisfied with the reception in 66.1% of cases and found the waiting time at the consultation inconvenient in 9.8% of cases (Table I).

**Table I:** Satisfaction from reception to diagnosis

	Very satisfied	Satisfied	Poor	Very Poor
Reception in the department	0	74 (66,1 %)	24 (21,4 %)	14 (12,5 %)
Waiting for the consultation	24 (21,5 %)	77 (68,7 %)	11 (9,8 %)	0
Information on pathology	34 (30,3 %)	74 (66,1 %)	4 (3,6 %)	0

From the preoperative examination to the day before surgery, patients were satisfied with the surgical staff and the quality of medical information in 66.1% and 71.4% of cases, respectively (Table II).

**Table II:** Satisfaction with preoperative examination (POE) up to the day before surgery

	Very satisfied	Satisfied	Poor	Very Poor
POE formalities	0	74 (66,1 %)	38 (33,9 %)	0
Pre-anaesthetic consultation	10 (8,9 %)	88 (78,5 %)	14(12,6 %)	0
Surgical staff	21 (18,7 %)	74 (66,1 %)	17(15,2 %)	0
Quality of information about the operation	26 (23,2 %)	80 (71,5 %)	6 (5,3 %)	0
Purchase of pre-operative prescriptions	14 (12,5 %)	53 (47,3 %)	45 (40,2 %)	0
Reception the day before surgery	18 (16,1 %)	85 (75,9 %)	9 (8 %)	0
Quality of hospital room	2(1,8 %)	24 (21,4 %)	61 (54,5 %)	25 (22,3 %)
Quality of bathroom facilities	5 (4,5 %)	32 (28,6 %)	50 (44,6 %)	25 (22,3 %)

From the day of surgery to the end of hospitalization, 16.1% of patients were very satisfied with the behavior of the medical-surgical team in the OR, while 17.9% were dissatisfied (Table III). Regarding discharge procedures, 71.4% and 80.4% of patients, respectively, were satisfied with the information given on discharge prescription and postoperative follow-up (Table IV).

**Table III:** Satisfaction from day of surgery to end of hospital stay

	Very satisfied	Satisfied	Poor	Very Poor
Team identification	8(7,2 %)	38 (33,9 %)	66 (58,9 %)	0
Respect for privacy	13 (11,6 %)	72 (64,3 %)	27 (24,1 %)	0
Confidentiality of information	18 (16,1 %)	80 (71,4 %)	12 (10,7 %)	2 (1,8 %)
Waiting time before entering the operating room	16 (14,3 %)	90 (80,4 %)	6 (5,3 %)	0
Team behavior in the operating room	18 (16 %)	54 (48,2 %)	20 (17,9 %)	20 (17,9 %)
Quality of the operating room	8 (7,1 %)	29 (25,9 %)	61 (54,5 %)	14 (12,5 %)
Quality of visits	32 (28,6 %)	66 (58,9 %)	14 (12,5 %)	0
Attitude of nurses	24 (21,4 %)	66 (58,9 %)	22 (19,7 %)	0
Attitude of surface technicians	0	77 (68,7 %)	29 (25,9 %)	6(5,4 %)
Attitude of on-call team	19 (17 %)	80 (71,4 %)	13 (11,6 %)	0

**Table IV:** : Satisfaction from day of surgery to end of hospital stay

	Very satisfied	Satisfied	Poor	Very Poor
Information on discharge orders	27 (24,1 %)	80 (71,2 %)	5 (4,47 %)	0
Follow-up information	21 (18,7 %)	90 (80,4 %)	1 (0,9 %)	0
Administrative formalities	18 (16,1 %)	90 (80,3 %)	4 (3,6 %)	0
Assessment of surgery	40 (35,7 %)	72 (64,3 %)	0	0

## Discussion

One of the biases of our study is the absence and ignorance of the inpatient charter, which leads to false assessments of the service provided by medical personnel by patients who have no reference to judge. The result is false satisfaction and false dissatisfaction. Other biases include the fact that patients have regained their health, which may have led to exaggerated positive evaluations. Patients from rural areas, who generally do not make demands, are sometimes satisfied with everything. These biases are common to most studies in the literature [10]. Other limitations of the study are the small sample size, the use of French language in the survey form, which required extensive explanations to respondents with a poor level of French, and the desire of some to abstain because they thought they were making a false judgment that could harm the health structure.

More than a third of patients were dissatisfied with the welcome they received on the ward, mainly due to the unfriendliness of the nursing personnel. Welcoming a patient is more than just a normal part of daily life. It's a special time for them, a time when they are sensitive and vulnerable and need someone to hold them [10]. The training and information sessions organized by the Togo Union of Hospital Practitioners will help to remedy this shortcoming. It is important to make patients feel welcome, as treatment begins as soon as they are admitted.

In 40.2% of the cases, the procedure for filling preoperative prescriptions was perceived as inadequate. In fact, it is the patient himself who goes from pharmacy to pharmacy to obtain the prescribed drugs and materials, as the former often do not have the full range prescribed. Preprepared kits of anesthetic and surgical drugs for standard procedures could help to overcome this difficult procedure.

The National Patient Safety Foundation in the United States has defined patient safety as the avoidance, prevention, and mitigation of adverse outcomes or injuries resulting from health care processes [4]. The presentation to surgical staff is consistent with this definition. It was rated as very satisfactory by 80% of patients. To minimize intraoperative incidents and accidents, some authors recommend a surgical staff meeting to discuss the case before surgery [9]. In our case, all patients are systematically scheduled for a surgical staff meeting, except in emergencies. This allows the team to confirm the diagnosis, reiterate the surgical procedure to the patient, and train the physicians in the specialty. During this presentation, some patients were unhappy with the impressive number of doctors and the length of time they had to wait before the meeting.

In 71.4% of the cases, the patients were satisfied with the confidentiality and the quality of the information provided on their pathology. It is in accordance with articles 192 and 196 of the Togolese Public Health Code, which stipulate that *“doctors are bound by professional secrecy (…)”* and that “all *patients are free to consult the doctor of their choice. The latter must inform the patient of his state of health, of the care he is to receive and of the possible consequences for him”* [6]. Despite the conditions of hospitalization in the ENT department, i.e. hospitalization in a common ward with beds not separated by a barrier, and also the number of patients per consultation, it is possible and necessary to respect professional secrecy and to explain in detail the diagnosis and treatment of the patient. This is particularly important in the case of surgical management. Staff behavior in the OR was rated poor and very poor 17.9% of the time. Indeed, in addition to the department's own OR medical-surgical team, the OR was staffed by undergraduate students in surgical instrumentation, anesthesia and critical care, as well as physicians studying for board certification. A large number of personnel can cause anxiety in patients. Furthermore, open communication in the OR, i.e., reminders to follow certain rules, protests against inappropriate behavior, and remarks during the preoperative period, frightens the patient [5, 7]. Chattering and inappropriate remarks between the members of the medical-surgical team during induction of anesthesia are poorly perceived by the patient on the operating table.

## Conclusion

This study shows that dissatisfaction exists at all levels, administrative and medical. At the end of this study, we recommend scrupulous compliance with the Code of Medical Ethics and the Hospital Patient Charter in order to satisfy all patients admitted to a health care facility. A prospective study will be carried out on a larger sample, taking into account the Patient Charter. This will allow a more in-depth assessment of satisfaction with the quality of care in our department.

## Funding

No funding was provided for this work.

## Authors’ contributions

Essobiziou AMANA, Winga FOMA and Guemessou NASSOU: data collection and writing of the article. Gérémie ANANIDJIN, Bathokédéou AMANA: revision of the article.

## Conflicts of interest

The authors declare no conflict of interests.
